# Protocol for Acupuncture Treatment of Lateral Elbow Pain: A Multisite Randomised Controlled Trial in China, Hong Kong, Australia, and Italy

**DOI:** 10.1155/2016/1868659

**Published:** 2016-11-23

**Authors:** Christopher Zaslawski, Christine Berle, Marcus Gadau, Wei Hong Li, Tie Li, Fu Chun Wang, Sergio Bangrazi, Lei Li, Stefano Liguori, Yan Song Liu, Yuan Sheng Tan, Shi Ping Zhang

**Affiliations:** ^1^Chinese Medicine, School of Life Sciences, University of Technology Sydney, Sydney, NSW, Australia; ^2^School of Chinese Medicine, Hong Kong Baptist University, Kowloon, Hong Kong; ^3^Changchun University of Chinese Medicine, Changchun, China; ^4^Paracelso Institute, Rome, Italy; ^5^World Federation of Acupuncture and Moxibustion Societies, Beijing, China

## Abstract

*Background*. Lateral elbow pain is one of the most common musculoskeletal pains associated with the upper limb and has an estimated population incidence of 1–3%.* Methods/Design*. This study protocol is for a multisite randomised controlled study and is designed to evaluate the clinical efficacy of acupuncture in the treatment of chronic (over three months' duration) lateral elbow pain. Four study sites, in the People's Republic of China, Hong Kong, Italy, and Australia, will recruit 24 participants each. A total of 96 participants will be randomised to either an acupuncture group or a sham laser control group. The primary outcome measure will be the Disabilities of Arm, Shoulder, and Hand questionnaire with secondary outcome measures of Pain-Free Grip Strength Test, Muscle Tension Test, and a pain visual analogue scale.* Discussion*. Key features for conducting a multisite international acupuncture randomised clinical trial have been detailed in this protocol.* Trial Registration*. This trial is registered at Australian and New Zealand Clinical Trial Registry ACTRN12613001138774 on 11 October, 2013.

## 1. Introduction

Lateral elbow pain (LEP, frequently but incorrectly termed lateral epicondylitis or tennis elbow) is one of the most common musculoskeletal pains associated with the upper limb and has an estimated population incidence of 1–3% [1]. It is a painful condition that is associated with the degeneration in the area of the common extensor tendon of the forearm. Histopathological findings have shown it to be a degenerative condition (tendinosis) of the common extensor tendon, with the extensor carpi radialis brevis tendon more commonly implicated as the primary location of tendinosis. The anatomic basis of the injury to the extensor carpi radialis brevis origin appears to be multifaceted, involving hypovascular zones, eccentric tendon stresses, and a microscopic degenerative response [2].

Optimum treatment of LEP is still debated and research has not been able to determine an optimum treatment [3]. Acupuncture has become increasingly recognised as an alternative treatment for pain, including LEP [4]. In a publication by the United States National Institutes of Health, it was determined that acupuncture may be useful as an adjunct treatment or an acceptable alternative treatment for tennis elbow (lateral elbow tendinosis) [5]. In the past 15 years, acupuncture has become increasingly recognised as an alternative treatment for pain, including pain from lateral elbow tendinosis. However, there is inconclusive evidence that needle acupuncture has a specific action in the treatment of this debilitating condition as shown by Gadau et al. in the latest systematic review on the topic due to low methodological quality and the small sample sizes of the individual studies [6]. The result from a nonrandomised controlled pilot study (*n* = 40) from our group suggests that acupuncture may be helpful in improving function and pain associated with LEP [7]. In this study, the treatment group (*n* = 20) received nine unilateral acupuncture treatments at LI 10 and LI 11 on the affected side with manual needle manipulation while the control group (*n* = 20) received nine sham-laser-acupuncture at the same acupoints. Measures included (i) disabilities of the arm, shoulder, and hand (DASH) questionnaire, (ii) pain-free grip strength (PFGS), and (iii) visual analogue scale (VAS) for pain. Significant differences for the DASH score, PFGS, and VAS between treatment and control group were found after the 8th treatment (*p* < 0.05). Only the DASH scores showed a significant difference between treatment and control group for all time points when measurements were taken which appears to be a sensitive and appropriate primary outcome measure. Results from this pilot study provided relevant information concerning treatment efficacy, credibility of control treatment, and sensitivity of different outcome measures and were subsequently used to develop this protocol.

Furthermore, there have been few international multisite randomised controlled acupuncture trials [8]. This protocol introduces some of the strategies and procedures that have been developed to standardise and remote monitor across sites to ensure the specific key procedures are conducted in accord with the protocol. This randomised clinical trial is designed to evaluate the efficacy of acupuncture in the treatment of chronic (over three months' duration) lateral elbow pain, with specific attentions to overcome some of the limitations of previous studies. The specific hypothesis to be tested in the study is whether acupuncture is more beneficial in improving physical function and reducing symptoms for people with chronic lateral elbow pain of over three months' duration compared to sham laser.

## 2. Methods and Design

### 2.1. Trial Location

The study is to be undertaken at four international sites. These are Changchun University of Chinese Medicine, People's Republic of China; Hong Kong Baptist University, Hong Kong SAR; Paracelso Institute, Italy; and the University of Technology Sydney, Australia. The study will be administered at outpatient clinics attached to each institution. The World Federation of Acupuncture/Moxibustion Societies (WFAS) will coordinate the trial and will be responsible for convening the face to face meetings.

### 2.2. Design

It is a prospective trial that is randomised, controlled, participant/outcome assessor and statistician blinded. It is a multicentre trial with two parallel groups (*n* = 24 at each site, total participants *n* = 96) with a primary endpoint evaluation at completion of the intervention phase (week 3) and a follow-up secondary evaluation three weeks later at week 6. The treatment group will receive traditional Chinese style acupuncture and the control group will receive inactive sham laser therapy to the same local acupuncture points as the treatment group using a modified inactive laser unit.

### 2.3. Randomisation

Randomisation will be performed with a 1 : 1 allocation using a computer generated sequence with the randomisation software Minim [10]. Minim is a program for randomising patients to treatment groups in clinical trials by the method of minimisation, which reduces the differences between the groups for the variables used in the allocation process. Stratification during randomisation will be used based on the participant's gender, age, and the DASH score initially recorded on intake form on recruitment into the study. Allocation concealment will involve a unique three-digit code number known only to the third party randomiser and the practitioner (not outcome evaluator or statistician).

### 2.4. Inclusion/Exclusion Criteria

To be included in the study, participants should have chronic lateral elbow pain (duration > 3 months); have unilateral localization of the pain; be between 18 and 80 years of age.

Participants will be excluded if they have a disease of the central or peripheral nervous system; have an inflammatory rheumatic disease; have gout; had an earlier episode of lateral elbow pain treated surgically; have had acupuncture treatment or physiotherapy for tennis elbow within the previous three months or acupuncture treatment for any problems within the previous week or concurrent physiotherapy for tennis elbow.

**Figure 1:**
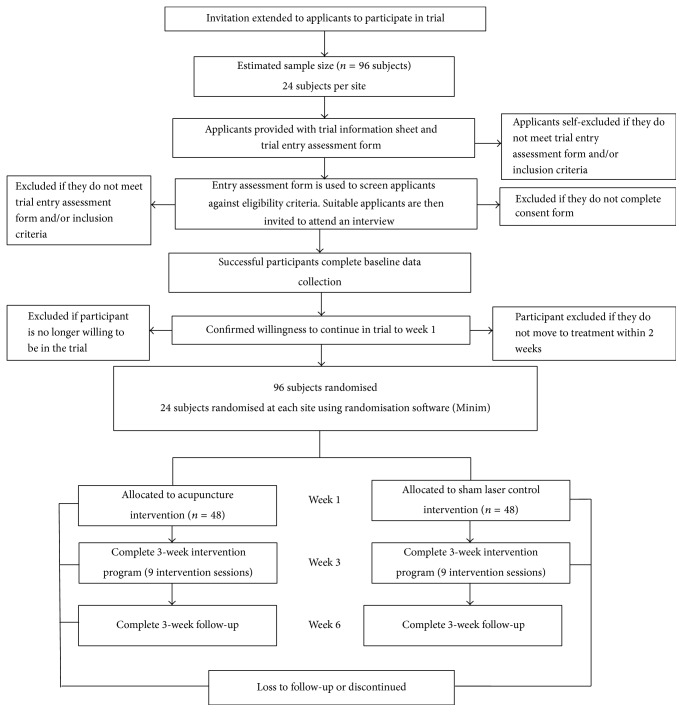
Diagram showing flow of participants.

Figure 1 shows the flow of participants through the study.

## 3. Interventions

The acupuncture administered is based on traditional Chinese medicine (TCM) meridian theory. Two preparative meetings of representatives from our study group had been held to discuss the study protocol, especially the selection of acupuncture and control interventions. Taking into account results of the pilot study, standardisation, and improvement from some previous methodological limitations, a standardised intervention procedure has been developed for both the acupuncture treatment and sham laser control groups. Interventions are not varied for either group; all participants will receive the same treatment at all sessions for their group. Only the affected arm is treated. Practitioners are required to apply a standardised intervention procedure to participants dependent on the participant's randomisation status. Acupoint selection was based on a consensus by all four recruiting centres following positive outcomes from the analysis of data generated from a pilot study undertaken by one of the research groups at Changchun University of Chinese Medicine [7].

All acupoints are to be marked with a semipermanent ink. The two acupoints are to be located as described in the* WHO Standard Acupuncture Point Locations in the Western Pacific Region* [11]. The acupoint Large Intestine (LI) 11 (Quchi) is located on the lateral aspect of the elbow, at the midpoint of the line connecting Lung 5 (Chizi) with the lateral epicondyle of the humerus. The acupoint LI 10 (Shousanli) is located on a line connecting LI 5 (Yangxi) (on the posterolateral aspect of the wrist, at the radial side of the dorsal wrist crease, distal to the radial styloid process, in the depression of the anatomical snuffbox) with LI 11 (Quchi), 2 body units (cun) inferior to the cubital crease on the forearm. The acupoint LI 10 will be located using a piece of elastic divided into 12 equal proportions to assist accurate proportional measurement [12].

### 3.1. Acupuncture

Single-use, stainless steel, presterile filiform 0.30 mm × 40 mm Hua Tuo brand needles will be used to needle the acupoint sites following cleansing of the skin with rubbing alcohol.

### 3.2. Needle Manipulation

The acupoint LI 11 will be needled first, perpendicular deep insertion up to 1.5 body units (cun), and then withdrawn to 1 cun depth. Following insertion, the traditional needle technique of “Wagging the Dragon's Tail” (WDT) will be performed. This involves holding the needle on distal end of the handle and bending it 45° left and then right with a speed of 1 Hz for 2 minutes or to the participant's tolerance. Then, the acupoint LI 10 will then be needled using oblique insertion at 45 degrees angled towards the elbow and the same needle technique of WDT applied for the same period of time (2 minutes) or to the patient's tolerance. Whenever the duration of needle manipulation is less than the required time of 2 minutes (due to patient request to stop due to excessive pain or sensation), a note will be kept of the time the technique was applied. Needling sensation (deqi) will be sought on both acupoints. Muscle twitch will be recorded on participant's daily reporting sheet if achieved on acupoints LI 10 and/or LI 11.

The needles will be retained for a further period of 24 minutes and then the process of needle manipulation will be repeated at each site and needles will be withdrawn. In total, the needles will remain in situ for 28 minutes. Nine acupuncture treatments will be administered over a three-week period (three treatments per week).

### 3.3. Online Training Video for Acupuncturists

A video of the needle technique (needle insertion and the technique of “Wagging the Dragon's Tail”) has been uploaded onto the cloud repository for review by all the needling research teams. This is to assist in standardising the intervention needling technique. All practitioners of needling had at least seven years of clinical experience and were credentialed as registered or licensed practitioners.

### 3.4. Control Intervention

The control group will receive inactive sham laser therapy to the same local acupuncture points as the treatment group using a modified inactive laser unit. This was chosen as the control as the inactive device appears to treat acupuncture points with laser light but is inert and does not puncture the skin [9]. The laser probe is rested lightly on the skin and the inactive sham laser will be applied at the same sites for the same duration and order as the acupuncture followed by a rest period of 24 minutes and then a further two minutes of sham radiation to the two acupoints will be administered. Similar to the acupuncture intervention, nine laser sessions will be administered over a three-week period (three treatments per week).

The laser unit will have the laser diode removed. Although the models of the machines will be different between different sites, the following properties will apply: The sham laser must have either light or sound to indicate functionality and be made of stand and probe; when the beam is invisible, it should be referred to as infrared; participants should be warned about the “harmfulness” of laser to the eyes; laser should be held away from their eyes; the trial participants as well as the practitioner should wear special laser-protection glasses.

### 3.5. Outcomes

The primary outcome is the Disabilities of Arm Shoulder and Hand (DASH) questionnaire. This is a 30-item questionnaire with descriptors for each question ranging between 1 (no difficulty) to 5 (unable) plus two extra modules for work and sport [13]. The DASH will be administered prior to intervention sessions one and nine and during the follow-up session at week six.

Secondary outcomes measures are as follows:

1. Pain-Free Grip Strength Test (PFGS) Metric measurement: all four sites will use a hand held dynamometer (model J00105 JAMAR). A mean of three measures will be used for analysis.
2. Muscle Tension Test (MTT) Metric measurement: all four sites will use the Lafayette manual muscle tester. A mean of three measures will be used for analysis.
3. Pain questionnaire-visual analogue scale (VAS): this is a 100 mm line with the descriptors “no pain” marked at one end of the scale and “excruciating” at the other. Three assessments will be made by the participant of the pain at rest, in motion, and during exertion.

In addition, the Massachusetts Acupuncture Sensation Scale (MASS) De Qi Questionnaire [14] which is a self-report 17-item questionnaire that uses a Likert scale will be administered twice following intervention sessions one and nine to measure needling sensation for both the acupuncture and sham laser groups.

All forms including outcome measure instruments (DASH, MASS, VAS, PFGS, and MTT) as well as all CRFs were translated into Chinese language by one of the researchers (WHL) and checked by two other fluent English/Chinese members of the research team (MG and ZSP) for use in Hong Kong and China.

All outcome measures will be assessed at baseline, week 3 (following completion of all nine intervention sessions), and at the follow-up session in week 9. The DASH questionnaire, PFGS, MTT, and VAS will be administered before the intervention session commences while the MASS will be completed following the intervention session.

### 3.6. Intervention Credibility I and II and Treatment Satisfaction Questionnaires

In addition, intervention credibility questionnaires (I at commencement and II at completion of the intervention phase) are to be administered to all subjects to assess levels of satisfaction with their allocated treatment. Questionnaire I asks “how confident are you that this treatment can alleviate your complaint?” and “how logical does this treatment seem to you?” [15]. A seven-point Likert scale (0–6) is to be used with each end point labelled “not confident” and “confident” to the first question and “not logical” and “logical” to the second question. This questionnaire is to be administered after allocation to an intervention group and prior to receiving their first treatment of acupuncture or sham laser. Questionnaire II askes “how confident would you be in recommending this treatment to a friend who suffered the same complaint?” and “how successful do you think this treatment would be in alleviating other complaints?” Similar to the intervention credibility questionnaire, a seven-point Likert scale (0–6) will be used with end point labelled “not confident” and “confident” to the first question and “not successful” and “successful” to the second question. This questionnaire is to be administered following the participants last intervention.

At the completion of the trial, a Treatment Satisfaction Questionnaire will also be administered. It includes two questions: “Do you believe you received a valuable therapeutic treatment for your lateral elbow pain?” (“yes,” “no,” or “do not know”) and “what are your reasons for believing this?”

**Figure 2:**
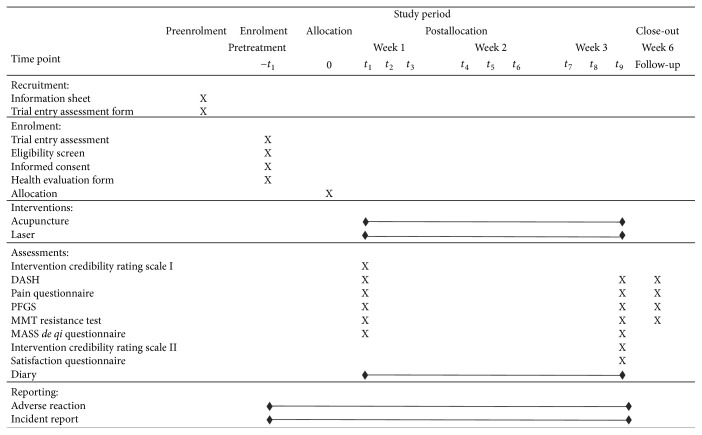
Schedule of enrolment, interventions, and outcome measures.

Figure 2 shows the schedule of enrolment, interventions, and outcome measures.

### 3.7. Trial Registration

The trial was registered at the Australian and New Zealand Clinical Trial Registry at http://www.ANZCTR.org.au/ACTRN12613001138774.aspx on the 11th October, 2013, following approval from each of the four institution's ethical approval committees (commonly referred to as Independent Research Boards) at all four sites.

### 3.8. Funding and Ethics Approval

It was agreed that each site will fund its own costs independently and seek human research ethics clearance from their regional human research ethics committee.

### 3.9. Strategies to Improve Participant Adherence

Upon successful admission to the trial, participants will be given a diary which monitors activities and adherence to the study criteria. Participants will be instructed on how to use the diary and an explanation for its use at their initial interview.

### 3.10. Site Monitoring

Each site will digitally video record specific segments of trial processes which will then be uploaded to a secure internet repository for checking against an agreed checklist by clinical trial personnel at the three other sites. The checklist to be used for checking each item of a procedure is shown as follows. A Daily Intervention Reporting Form is to be used to monitor any comments or changes reported by participants. An Adverse Reaction Report Form will be used to record and monitor any adverse events to the interventions and an Incident Report Sheet has been developed to record any injury or incident which affects any person participating in the trail which was not a reaction to the prescribed interventions.

The nine items and their subelements of the video monitor checklist are as follows:

1. Recruitment guidelines
  Use of information sheet
  Meeting recruitment numbers
2. Interview
  Recruitment officer being familiar with the trial protocol and able to answer applicant's questions appropriately
  Trial entry assessment form completed fully
  Enrolled applicants meeting eligibility criteria
  Consent forms completed
  Consent forms stored appropriately and separated to participant's folder
3. Randomisation
  Randomisation officer using MINIM software correctly
  Randomisation officer stratifying as per allocation and randomisation protocol
4. Baseline and ongoing data collection
  Participant deidentified folders and documentation developed and stored securely
  Assessor using correct procedures in regard to scheduling of appointments, interventions, and assessments in accord with the protocol
  Assessor being familiar with questionnaires and their administration
  Assessor being familiar, reliable, and consistent with physical testing equipment
  Competence and consistent in recording test outcomes on appropriate CRFs
5. Intervention
  Ensuring that practitioners be able to adequately measure and mark up acupoints on the participant for treatment in accord with protocol
  Participant positioned appropriately
  Practitioners having been trained and each intervention (acupuncture and laser) being delivered correctly
  Practitioners knowing the protocol for any adverse reactions and/or how to report an incident
6. Data collection
  Folders being complete with all deidentified files
  Participant folders stored in a secure place
7. Data entry
  All CRFs having been collected
  Data entry personnel being competent in how to code CRFs for data entry
  Security of both completed paper forms and electronic spread sheets, including logon and link password protection to ensure no data tampering
  Software virus scanning
  Random data checking (audit) to ensure that data has been entered correctly
  Regular back-up of database
8. Participant retention and follow-up
  Participants having attended all treatments and if not, the recruitment officer has followed up
9. Meet and interview
  Trial coordinator of site and at least one clinician, trial personnel, or coordinator
    Observing at least two participants being treated
    Verifing that all adverse events have been documented and recorded on the correct forms

### 3.11. Trial and Data Management

All trial documents including case report forms, outcome measures, the trial protocol, digital video clips required for trial monitoring, and the final data excel spreadsheets will be uploaded into a secure digital cloud repository site which will be managed by the Hong Kong trial personnel. Regular teleconferences involving all sites will be undertaken on a regular basis and conducted in both English and Chinese language. Minutes of the teleconference meetings will be published and uploaded into the digital cloud repository site.

### 3.12. Statistical Evaluation

The statistician will be blind to allocation status and a variety of statistical tests including descriptive and inferential tests (ANOVA, chi square) will be used. A blind third party at the Hong Kong site will undertake the statistical analysis following dual data entry, data checking, and revision. Sample size (*n* = 96, 24 subjects for each site) was based on the China pilot study data associated with the primary outcome, the DASH questionnaire [7] (Liu et al., 2016).

### 3.13. Protocol Preparation

The initial trial protocol was developed in English by the Australian clinical trial personnel following consensus by all trial sites. The protocol was jointly translated by the Australian and Hong Kong clinical trial personnel into Chinese for use by the Hong Kong and Changchun trial sites. This protocol has been prepared in accordance with the SPIRIT (Standard Protocol Items: Recommendations for Interventional Trials) 2013 [16] and also conforms to the requirements of the Revised STandards for Reporting Interventions in Clinical Trials of Acupuncture (STRICTA) 2010 [17] (see Table 1).

### 3.14. Current Status

Each site has completed data collection and double entry data sheets have been uploaded to the digital repository for analysis which has been conducted.

## 4. Discussion

This is the first time to our knowledge that a protocol for a multisite international acupuncture randomised clinical trial has been developed in English. There were many challenges in designing the study, developing the protocol, and operationalizing the trial. Issues such as language and communication, achieving protocol consensus, trilingual case report forms and outcome measures, trial monitoring, individual site funding, and ethical approval processes needed to be overcome.

As an international study in different countries with different cultures, one of the first issues was finding a common language. Because several researchers at two sites were bilingual, with English being a common language, the two main languages used for communication and CRF development were English and Chinese. However, because enrolled subjects in each of the countries would not be bilingual, all documents including the protocol, CRFs, and outcome measures had to be developed in the English language and translated into Chinese (for Hong Kong and Chinese subjects) and Italian (Italian subjects) language.

Achieving consensus for the protocol and the intervention also required careful reflection and planning. Several face to face meetings were held prior to designing the study to develop the protocol and define the interventions. One of the research partners from the Changchun University of Chinese Medicine (WFC) had had extensive experience in treating lateral elbow pain and suggested a simple acupuncture technique consisting of two local acupoints (Large Intestine 10 and Large Intestine 11) and a specific needle technique (Wagging the Dragon's Tail). The research partners decided that a pilot study would be necessary to ensure that the technique was efficient and asked the Chinese research group to conduct a pilot study before incorporating the acupuncture technique [7].

Another unique aspect to the protocol was site monitoring. Due to the international location of the four sites, a remote monitoring procedure was developed. This involved developing a checklist of items to ensure the trial was being conducted correctly (as per protocol) and in a standardised manner. The monitoring checklist consisted of several categories each assessing a different aspect of the trial. These categories were recruitment guidelines (2 items); interviews (5 items); randomisation (2 items); baseline and ongoing data collection (5 items); interventions (4 items); data collection (2 items); data entry (6 items); and participant retention and follow-up (1 item).

Each research site is required to make short digital video clips of segments of the study procedures and upload them into the digital cloud to a secure repository for viewing and assessment by the research partners. Each site can then make an assessment of each item as being present or absent and is able to provide feedback to each study group so that revision can be made, if necessary.

Another consideration was funding. Given the different resources and capabilities at each site, it was decided at the onset that each site should be responsible for funding and the supply of both human and physical resources for their component of the trial. Finally, human research ethics approval was sought at each individual site using the standard CRFs and a common consent and information sheet. Special attention was given to the wording used for the sham laser control in the consent form; prior to applying for ethics approval, each site agreed to keep certain clauses similar in the consent form such as “I understand that the purpose of this study is to identify if there are any health benefits using acupuncture or laser for people with lateral elbow pain” and “Due to the design of the study I will not receive any information on the specific goals of the interventions.” This second clause was to address the issue of full informed consent as the subjects were not aware that a sham laser is to be administered. If they were fully informed as to this fact, there is a strong likelihood they may become aware they are to receive a placebo sham laser treatment. The argument for not fully informing participants was based on the lack of a gold standard for treatment of LEP and was that withholding other treatments will not be detrimental to the time course or progression of the condition. Human ethical approval was sought and obtained for all sites in each jurisdiction.

It is anticipated that the trial results are to be published in early 2017.

## Figures and Tables

**Table 1:** Revised STandards for Reporting Interventions in Clinical Trials of Acupuncture (STRICTA) 2010 items and their description in the protocol for the treatment group.

Item	Detail	Description of item
(1) Acupuncture rationale	(1a) Style of acupuncture (e.g., Traditional Chinese medicine, Japanese, Korean, Western medical, Five Element, ear acupuncture, etc.)	Traditional Chinese medicine
	(1b) Reasoning for treatment provided, based on historical context, literature sources, and/or consensus methods, with references where appropriate	Consensus by all four recruiting centres following positive outcomes from the analysis of data collected from a pilot study
	(1c) Extent to which treatment was varied	Not varied except manipulation time shortened when patient by patient

(2) Details of needling	(2a) Number of needle insertions per subject per session (mean and range where relevant)	Two filiform needle insertions per subject used per session
	(2b) Names (or location if no standard name) of points used (uni/bilateral)	Large Intestine 11 (*Quchi*), unilateral Large Intestine 10 (*Shousanli*) unilaterally
	(2c) Depth of insertion, based on a specified unit of measurement or on a particular tissue level	Needling up to 1.5 body units (cun)
	(2d) Response sought (e.g., *de qi* or muscle twitch response)	Deqi measured after intervention sessions 1 and 9 using MASS
	(2e) Needle stimulation (e.g., manual, electrical)	Manual needle stimulation standardised: Wagging the Dragon's Tail
	(2f) Needle retention time	Needles retained 28 minutes
	(2g) Needle type (diameter, length, and manufacturer or material)	Single use, presterile, filiform needles 0.30 mm × 40 mm *Hua Tuo* brand needles (Suzhou Needle Company)

(3) Treatment regimen	(3a) Number of treatment sessions	Nine sessions
	(3b) Frequency and duration of treatment sessions	Three treatments per week

(4) Other components of treatment	(4a) Details of other interventions administered to the acupuncture group (e.g., moxibustion, cupping, herbs, exercises, and lifestyle advice)	No additional interventions administered Medication use logged in diary if used
	(4b) Setting and context of treatment, including instructions to practitioners, and information and explanations to patients	Four sites in China, Changchun University of Chinese Medicine hospital clinic; Hong Kong, Hong Kong Baptist University clinic; Italy (Center on Nonconventional Medicine clinic); and Australia (UTS TCM Clinic)

(5) Practitioner background	(5) Description of participating acupuncturists (qualification or professional affiliation, years in acupuncture practice, other relevant experience)	All practitioners had at least seven years of clinical experience and were members of the World Federation of Acupuncture-Moxibustion Societies (WFAS).

(6) Control or comparator interventions	(6a) Rationale for the control or comparator in the context of the research question, with sources that justify this choice	Noninvasive inactive sham laser which does not pierce the skin See [9] for justification
	(6b) Precise description of the control or comparator. If sham acupuncture or any other type of acupuncture-like control is used, provide details as for items 1 to 3 above.	Inactive sham laser therapy to the same local acupuncture points as the treatment group using a modified inactive laser unit

## Abbreviations

CRF: Case report form
DASH: Questionnaire: disabilities of the arm, shoulder, and hand questionnaire
LEP: Lateral elbow pain
MASS: Massachusetts Acupuncture Sensation Scale
MTT: Muscle Tension Test
PFGS: Pain-Free Grip Strength
RCT: Randomised controlled trial
SPIRIT: Standard Protocol Items: Recommendations for Interventional Trials
STRICTA: Revised STandards for Reporting Interventions in Clinical Trials of Acupuncture
TCM: Traditional Chinese medicine
TEA-IS-CHAI: Tennis elbow acupuncture international study: China, Hong Kong, Australia, and Italy
VAS: Visual analogue scale
WDT: Wagging the Dragon's Tail
WFAS: World Federation of Acupuncture/Moxibustion Societies
WHO: World Health Organisation.
